# Fall Armyworm Gut Bacterial Diversity Associated with Different Developmental Stages, Environmental Habitats, and Diets

**DOI:** 10.3390/insects13090762

**Published:** 2022-08-24

**Authors:** Dan-Dan Li, Jin-Yang Li, Zu-Qing Hu, Tong-Xian Liu, Shi-Ze Zhang

**Affiliations:** State Key Laboratory of Crop Stress Biology for Arid Areas, College of Plant Protection, Northwest A&F University, Xianyang 712100, China

**Keywords:** *Spodoptera frugiperda*, gut microbiota, developmental stage, host diet, environmental habitat, 16S rRNA

## Abstract

**Simple Summary:**

Microorganisms play a crucial role during the growth and development of insects. However, as a major invasive pest, the diversity and dynamics of gut microbes with different developmental stages, environmental habitats, and diets in *Spodoptera frugiperda* remain unclear. The abundant gut microbes of *S. frugiperda* may be beneficial for its abilities of invasiveness and adaptation. Therefore, it is of great importance to systematically understand the microbial dynamics of *S. frugiperda*. This study systematically explored the changes of microorganisms of *S. frugiperda* at each developmental stage. Furthermore, the differences in gut microorganisms of *S. frugiperda* in different living environments (field and laboratory) and different foods (corn and artificial diet) were also explored. Our results suggest that *S. frugiperda* gut microbes vary greatly at different developmental stages and demonstrate vertical transmission of bacteria in *S. frugiperda*. Furthermore, environment and diet can also alter gut microbes. We performed a detailed investigation of the microbial community of *S. frugiperda* that provides a basis for future research. Since the plasticity of insect gut microbes helps insects utilize different foods and enhances insect fitness, a comprehensive understanding of *S. frugiperda*’s gut microbiome will help develop novel pest control strategies for this invasive pest prevention.

**Abstract:**

The fall armyworm, *Spodoptera frugiperda* (Lepidoptera: Noctuidae), is a major invasive pest that seriously threatens world agricultural production and food security. Microorganisms play a crucial role in the growth and development of insects. However, the diversity and dynamics of gut microbes with different developmental stages, environmental habitats, and diets in *S. frugiperda* remain unclear. In this study, we found the changes of the microbiome of *S. frugiperda* across their life stages, and the bacteria were dominated by Firmicutes and Proteobacteria. The community composition of the egg stage was quite different from other developmental stages, which had the highest community diversity and community richness, and was dominated by Proteobacteria. The bacterial community compositions of male and female adults were similar to those of early larvae stage (L1–L2), and operational taxonomic units (OTUs) with abundant content were *Enterococcus* and Enterobacteriaceae bacteria, including *Enterobacteria*, *Klebsiella*, *Pantoea*, and *Escherichia*. The third instar larvae (L3) mainly consist of *Enterococcus*. The late stage larvae (L4–L6) harbored high proportions of *Enterococcus*, *Rhodococcus,* and *Ralstonia*. There was no significant difference in gut microbial composition between field populations and laboratory populations in a short period of rearing time. However, after long-term laboratory feeding, the gut microbial diversity of *S. frugiperda* was significantly reduced. *Enterococcus* and *Rhodococccus* of *S. frugiperda* feeding on maize showed higher relative proportion, while the microbial community of *S. frugiperda* feeding on artificial diet was composed mainly of *Enterococcus*, with a total of 98% of the gut microbiota. The gene functions such as metabolism, cell growth and death, transport and catabolism, and environmental adaptation were more active in *S. frugiperda* feeding on corn than those feeding on artificial diet. In short, these results indicate that developmental stage, habitat, and diet can alter the gut bacteria of *S. frugiperda*, and suggest a vertical transmission route of bacteria in *S. frugiperda*. A comprehensive understanding of gut microbiome of *S. frugiperda* will help develop novel pest control strategies to manage this pest.

## 1. Introduction

Animal-microbial symbiosis is extremely important to the ecosystem [1]. Microbial symbionts are especially ubiquitous in insects, and they exist in insect exoskeletons, gut, and even within insect cells, which are usually beneficial or necessary for survival of insect hosts [2]. Insects can use microorganisms to enhance their life performance and adaptation to the various environmental changes [3]. Many insect-related microorganisms can not only provide specific nutrients that insects cannot synthesize themselves, such as essential amino acids [4,5] and B vitamins [6,7], but also protect their insect hosts against other invasive organisms, such as pathogens, parasitoids or predators [8,9,10]. In addition, symbiotic microorganisms can also enhance the resistance of insects to pesticides [11,12].

Many factors, including diet, life stage, and host habitat affect the structure of the gut microbial community [13,14,15,16]. In order to adapt to the different environmental changes, insects have evolved different composition of symbiotic microorganisms in the different developmental stages [14]. In principle, diet can influence the gut microbiota directly and indirectly [2,17]. For example, protein can lead to an increase in the abundance of specific microbiota in *Blattella germanica* [18]. Microbial communities of isogenic *Drosophila melanogaster* fed on different diets are different, but three distantly related Drosophilids fed on the same medium have similar bacterial microbiome [19].

The fall armyworm, *Spodoptera frugiperda* (Lepidoptera: Noctuidae) is a serious invasive insect pest. Due to its overeating major crops such as corn and rice, long-distance super migration and spreading ability, *S. frugiperda* was listed as one of the top 10 hazardous plant pests in the world by the CAB International (CABI) in 2017 (https://www.cabi.org/isc/fallarmyworm (accessed on 10 January 2021)). Moreover, *S. frugiperda* is posing a serious threat for potential economic losses to other staple crops such as wheat, soybean, cotton, tomato, and cabbage [20]. It is well known that insects have abundant and diverse gut microbes, and the microbiomes not only provide important nutrients for their insect hosts but also assist in the food digestion, immune defense, detoxification, and adaptation to changing environments [21,22]. The gut microbes Archaea and Bacteria play an important role in the nutritional requirement of the fifth instar larvae of *S. frugiperda* [23]. The analysis of the gut microbiota of susceptible, insecticide-resistant strains and field populations of *S. frugiperda* indicates that the gut microbes have a high diversity and the ability to metabolize insecticides in field populations of *S. frugiperda* [24]. Recently, Lv et al. [25] reported that the gut microbial community of the fifth instar larvae of *S. frugiperda* is significantly affected by different host species. However, the previous studies on the gut microbial community of *S. frugiperda* were limited to a certain stage of the host’s development and rarely investigated the changes of microorganisms throughout the complete life stages. Thus, diversity and dynamics of the bacterial community across different developmental stages of *S. frugiperda* are still unclear.

It is well known that altering the insect gut microbiome can influence insect behavior, which may lead to new approaches to pest control, but these depend largely on a detailed understanding of insect-associated microorganisms [2]. For example, the elimination of the symbiont *Symbiotaphrina kochi* in *Lasioderma serricorne* beetles depresses larval development [26]. Insect gut microbes are able to interact with the host, and then the high abundance bacteria are more likely to play an important role in host adaptation. In addition, previous studies have shown that the diet consumed, living environment, and developmental stage of insects may lead to the differences of gut microbial communities and dynamics [18,27,28]. As one of the 10 most notorious plant pests in the world, systematic study on the interaction between *S. frugiperda* and gut microbiome can not only provide a basis for in-depth understanding of its rapid adaptation in migration area, but also could provide a theoretical basis for the development of new control strategies and technology. However, to date, limited data have been available on *S. frugiperda* microbiota. We hypothesized that the rapid adaptation of *S. frugiperda* in the invasive areas may be related to the abundance of its gut microbiome. Therefore, in this study, we systematically explored the changes of microorganisms of *S. frugiperda* at each developmental stage, and the differences of gut microorganisms of *S. frugiperda* in different living environments (field and laboratory) and different foods (corn and artificial diet). The present work not only provides valuable information for a comprehensive understanding of gut microbiome across the life history of *S. frugiperda*, but also assists the development of novel pest control strategies for prevention of this invasive pest.

## 2. Materials and Methods

### 2.1. Rearing of S. frugiperda

Maize (*Zea mays* L. var. Shandan 636) seeds were purchased from Yangling Agricultural High-Tech Development Co., Ltd. (Yangling, China), and sown in plastic pots with a 3:1:1 mixture of commercial peat moss (Pindstrup Mosebrug A/S, Ryomgaard, Denmark), perlite and vermiculite in an artificial climate room (25–30 °C, 50–80% RH and a photoperiod of 16L:8D). The plants of 14 days old were used for the experiments. The *S. frugiperda* larvae were collected from maize field (34°17′37.01″ N, 108°01′03.34″ E) in Yangling, Shaanxi Province, in July 2019, and individually put into plastic boxes (4 × 3 × 3 cm) and then brought back to the lab for rearing with maize seedlings in climatic chambers (LRH-400A-G3, Zhujiang^®^, Guangdong THK Scientific Instrument Co., Ltd., Shaoguan, Guangdong, China) at 25 ± 1 °C, 50–80% relative humidity and a photoperiod of 16:8 h (L:D).

### 2.2. Experimental Design

For field populations (Field), the 5th instar larvae of *S. frugiperda* were collected in the field and then brought back to the lab for dissection of the whole gut. The lab population of *S. frugiperda* (Lab0) was collected from the same field and was reared with maize seedlings under laboratory conditions. Field and lab populations were used to compare the differences of gut microbiome of *S. frugiperda* in different environments. The Lab0 population was continuously raised for 10 generations (Lab10) under laboratory conditions to validate the shaping of the gut microbiome by the environment. The artificial diet (DF) and maize leaves (MF) were used in rearing the *S. frugiperda* to test the effect of the diet on the gut bacterial composition, respectively. Artificial diet was improved according to Prasanna et al. [29] and the main ingredients are as follows: 180 mL distilled water, 15 g soybean powder, 12 g wheat bran, 2 g casein, 4 g yeast powder, 1.2 g ascorbic acid, 4 g agar, 150 mg choline chloride, 300 mg sorbic acid, 35 mg inositol, 30 mg cholesterol, 750 mg methyl-p-hydroxybenzoate, 0.1 mL formaldehyde.

### 2.3. Collection of Tissue Samples and DNA Extraction

The surface of *S. frugiperda* larvae and adults was washed with 0.5% NaClO for 2 min, 75% ethanol for 1 min and rinsed three times with sterilized-deionized water [30]. Previous studies have shown that the entire gut can provide a more accurate assessment of gut microbial composition [31], so the whole gut of *S. frugiperda* larvae was used in this study. The gut tissue was dissected in 0.01 M phosphate-buffered solution (PBS; PH7.4) under a dissecting microscope (Nanjing Jiangnan Novel Optics Co., Ltd., Nanjing, China). Due to the small size of the early instar larvae and eggs of *S. frugiperda*, a large number of samples were required for sequencing. Gut tissue collection for each replication at different developmental stages: the first instar larvae (L1) sample contained 500 individuals, the second instar larvae (L2) sample contained 300 individuals, the third instar larvae (L3) sample contained 100 individuals, the fourth instar larvae (L4) sample contained 50 individuals, the fifth instar larvae (L5) and the sixth instar larvae (L6) sample contained 5 individuals, respectively, the male (Male) and female (Female) adult sample contained 20 adults, respectively. The whole egg was used for sampling and each replicate contained 500 eggs. In addition, the fifth instars of lab (Lab0 and Lab10) and field (Field) populations, and artificial diet-feeding (DF) and maize leaf-feeding (MF) populations were used to collect the gut tissue. Each treatment included 3 replicates. The dissected gut tissue samples were collected into the 1.5 mL tube and were immediately flash frozen in liquid nitrogen and stored at −80 °C. The total nucleic acid was extracted using the FastDNA^®^ SPIN Kit for Soil (MP Biomedicals, Qbiogene Inc., Carlsbad, CA, USA) following the manufacturer’s protocol. The sterile PBS without insect tissue was used as a negative control both in DNA extraction and PCR amplification to detect reagents and environmental contamination [31]. The integrity and quality of the extracted DNA were evaluated on 1% agarose gel electrophoresis and a NanoDrop^®^ ND-2000 spectrophotometer (Thermo Fisher Scientific, Wilmington, DE, USA), respectively [32].

### 2.4. Sequencing of 16S rRNA Gene

Targeted amplicons of the V3−V4 region of 16S rRNA gene were generated with primers 338F and 806R [33]. A 20 μL PCR reaction mixture contained 4 μL of 5× FastPfu Buffer, 2 μL of 2.5 mM dNTPs, 0.8 μL of Forward Primer (5 μM), 0.8 μL of Reverse Primer (5 μM), 0.4 μL of FastPfu Polymerase, 0.2 μL of BSA, and 10 ng of Template DNA. PCR amplification was conducted in ABI GeneAmp^®^ 9700 following the conditions: 3 min at 95 °C, followed by 30 cycles of 30 s at 95 °C; 30 s at 50 °C; 45 s at 72 °C, and 10 min at 72 °C. All samples were amplified in triplicate. The PCR product was extracted from 2% agarose gel and purified using the AxyPrep DNA Gel Extraction Kit (Axygen Biosciences, Union City, CA, USA) according to manufacturer’s instructions and quantified using Quantus™ Fluorometer (Promega, Madison, Madison, USA). Sequencing libraries were generated with TruSeq^TM^ DNA Sample Prep Kit (New England Biolabs, Ipswich, SD, USA) and were sequenced on the Illumina MiSeq PE300 platform (Illumina, San Diego, CA, USA). Sequencing was performed by the Shanghai Majorbio Bio-pharm Technology Co., Ltd. (Shanghai, China) Thirty-nine DNA samples were sequenced successfully.

### 2.5. Microbiome Analyses

The PE reads obtained by Miseq sequencing were spliced according to the overlap relationship, and then quality-filtered by fastp version 0.19.6 and merged by FLASH [34]. The number of mismatches allowed by barcode was 0, and the maximum number of primer mismatches was 2. Raw data of the sequence were analyzed using QIIME. Reads that could not be assembled were discarded. Sequences with 97% similarity were clustered as operational taxonomic units (OTUs) using UPARSE. Manually filter the OTU table, i.e., remove chloroplast and mitochondria sequences in all samples. To minimize the effects of sequencing depth on alpha and beta diversity measure, the number of 16S rRNA gene sequences from each sample was rarefied, which still yielded an average Good’s coverage of 99.09%, respectively. The classification of representative sequences for each OTU were analyzed using RDP Classifier against a 16S rRNA gene database (Silva v138) using a confidence threshold of 0.7. The microbiome function was predicted by PICRUSt2 based on OTU representative sequences. Bioinformatic analysis of the gut microbiota was carried out using the Majorbio Cloud platform (https://cloud.majorbio.com (accessed on 15 May 2022)). Based on taxonomic information, statistical analysis of community structure was performed at each classification level. On the basis of the above analysis, a series of in-depth statistical and visual analyses such as multivariate analysis and difference significance test were performed on the community composition and phylogenetic information of multiple samples. Alpha diversity including Chao1 richness, Ace index, Shannon index, and Simpson index were calculated with Mothur to investigate community diversity and community richness. The Unifrac distance matrices were constructed and visualized in principal coordinate analysis (PCoA). More details about the tools used are listed on Table S1.

### 2.6. Statistical Analysis

The PERMANOVA test was used to assess the percentage of variation explained by the treatment along with its statistical significance using Vegan v2.5–3 package. Statistical test of significance was performed for multiple (one-way ANOVA, LSD post hoc test) and two-group (Student’s *t*-test, *p* < 0.05) treatments to detect statistical changes in community structure between treatments. These differences were considered significant at *p* < 0.05 level. Data were analyzed by using statistical software package SPSS 20.0 (SPSS Inc., Chicago, IL, USA). A similarity analysis (ANOSIM) was performed on bacterial communities at different developmental stages and different treatment groups.

## 3. Results

### 3.1. Sequencing Data of 16S rRNA

Negative controls are key to identify potential contamination. In this study, no bacteria were detected in the negative control, and the contamination of environmental and reagent microorganisms was excluded. Data sequencing and analysis of 39 samples for studying diversity were completed, and a total of 1,697,034 optimized sequences and 719,118,584 bases were obtained, with an average sequence length of 423 bp. Sequencing data statistics of all samples are shown on Table S2. The rarefaction curves of all samples reached a plateau stage, indicating that the sample numbers of all samples were sufficient (Figure S1). At the phylum level, Firmicutes, Proteobacteria, Actinobacteriota, Cyanobacteria, and Chloroflexi were the top five phyla.

### 3.2. Gut Microbiota Composition of S. frugiperda across Different Developmental Stages

**Figure 1:**
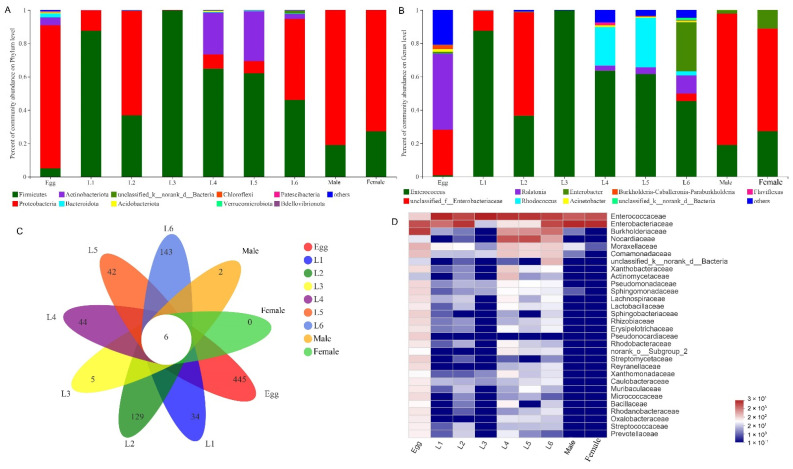
Gut bacterial community dynamics in different developmental life stages of *S. frugiperda*. (**A**) Gut bacteria composition at the phylum level; (**B**) Gut bacteria composition at the genus level; (**C**) Venn plot of OTUs in different developmental life stages; (**D**) Heatmap of the top 30 abundant families showing the relative abundance of the bacteria taxa assigned to a family level.

To investigate the variability of *S. frugiperda* bacterial communities at different developmental stages, we collected the samples of egg, L1–L6, and adult (male and female). Our results showed that the microbial diversity in the egg stage was the highest, and the microbial diversity decreased dramatically after the eggs hatched into larvae; in the larval stage, L6 had the highest microbial diversity; the adult stage had the lowest community richness (Figure S2). Firmicutes were the most abundant bacterial community of the larval stage; the dominant bacterial phylum in the egg and adult stages was Proteobacteria, followed by Firmicutes (Figure 1A). At the genus level, *Ralstonia* was the most abundant bacterium in the egg stage, followed by Enterobacteriaceae, including *Enterobacteria*, *Klebsiella*, *Pantoea*, and *Escherichia*; the bacterial community composition of male and female adults was similar to that of early larvae stage (L1–L2), and OTUs with abundant content were *Enterococcus* and Enterobacteriaceae bacteria, including *Enterobacteria*, *Klebsiella*, *Pantoea*, and *Escherichia*; the bacterial community of L3 mainly consisted of *Enterococcus*; the community composition of the late larvae (L4–L6) harbored high proportions of *Enterococcus*, *Rhodococcus,* and *Ralstonia* (Figure 1B).

**Figure 2:**
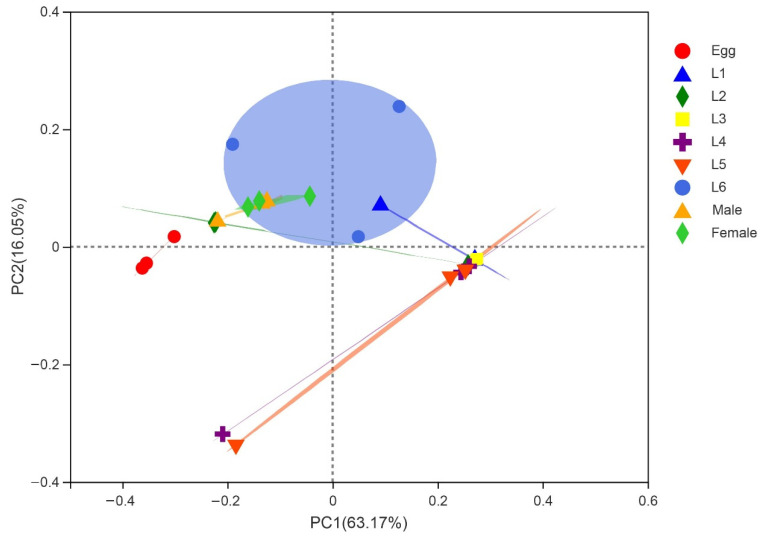
Principal coordinate analysis (PCoA) of community structure of different developmental life stages of *S. frugiperda*. Each symbol represents a sample.

The community heatmap analysis at family level allowed us to view the community composition in more details (Figure 1D). During the egg stage, the most abundant OTUs were Enterobacteriaceae and Burkholderiaceae. The bacterial community composition of male and female adults was similar to early larvae stage (L1–L2), and OTUs with abundant content were Enterococcaceae and Enterobacteriaceae. The dominant OTUs in the L3 were Enterococcaceae. The community composition of the late larvae (L4–L6) was similar, and the abundant OTUs were Enterococcaceae and Enterobacteriaceae, followed by Burkholderiaceae and Nocardiaceae. Among them, Enterococcaceae had a higher abundance at all developmental stages. PCoA based on the weighted unifrac distance showed that the samples from male and female adults were the most uniform, sharing similarities (Figure 2). The similarity analysis results indicated that there were significant differences in the bacterial community of *S. frugiperda* across developmental stages (ANOSIM: R = 0.533, *p* = 0.001; PERMANOVA: R = 0.061, *p* = 0.001).

### 3.3. Common and Unique Microbes among All Developmental Stages of S. frugiperda

Six OTUs, i.e., OTU478 (Proteobacteria, Enterobacteriaceae), OTU956 (Actinobacteriota, Corynebacteriaceae), OTU877 (Proteobacteria, Enterobacteriaceae), OTU346 (Firmicutes, Enterococcaceae), OTU784 (Firmicutes, Enterococcaceae), OTU884 (Proteobacteria, Moraxellaceae) were stable in different developmental stages of *S. frugiperda* (Figure 1C and Figure S3A; Table S3). The microbiomes of female adults had no additional OTUs, but male adults had two unique OTUs, i.e., OTU1446 (Rhodocyclaceae) and OTU1260 (Rikenellaceae); the egg stage had the largest number and diversity of unique OTUs, with the highest proportions being Desulfitobacteriaceae (10.75%), Clostridia (8.36%) and Thermoanaerobacteraceae (5.97%) (Figure S3B); the most abundant unique OTUs in L1 were Dojkabacteria (22.22%), 37–13 (16.67%) and Run-SP154 (16.67%) (Figure S3C); in L2, the most abundant unique OTUs were Cyanobiaceae (10.91%), Subgroup_7 (10.91%) and Pirellulaceae (9.09%) (Figure S3D); in L3, unique OTUs consisted of Desulfomicrobiaceae (75%) and Calditrichaceae (25%) (Figure S3E); in L4, the most abundant unique OTUs were 11–24 (34.24%), PHOS-HE36 (25.76%), and Magnetospirillaceae (17.97%) (Figure S3F); in L5, the most abundant unique OTUs were Petrotogaceae (49.45%), Marinobacteraceae (10.99%), and Desulfuromonadia (10.99%) (Figure S3G); in L6, the most abundant unique OTUs were Proteobacteria (17.97%), Hymenobacteraceae (15.63%), and Leptospirillaceae (10.16%) (Figure S3H).

PICRUST analysis predicted that “Metabolic pathways” and “Biosynthesis of secondary metabolites” were abundant in all developmental stages of *S. frugiperda*. Phosphotransferase system (PTS) was more abundant in larvae and adults than in eggs. PTS mainly phosphorylates various sugars and their derivatives through the phosphorylation cascade and then transports them into the cell. Starch and sucrose metabolism were more abundant in larvae than in adults and eggs (Figure S4).

### 3.4. Comparison of Gut Bacterial Communities of S. frugiperda Associated with Different Environmental Habitats of Host

**Figure 3:**
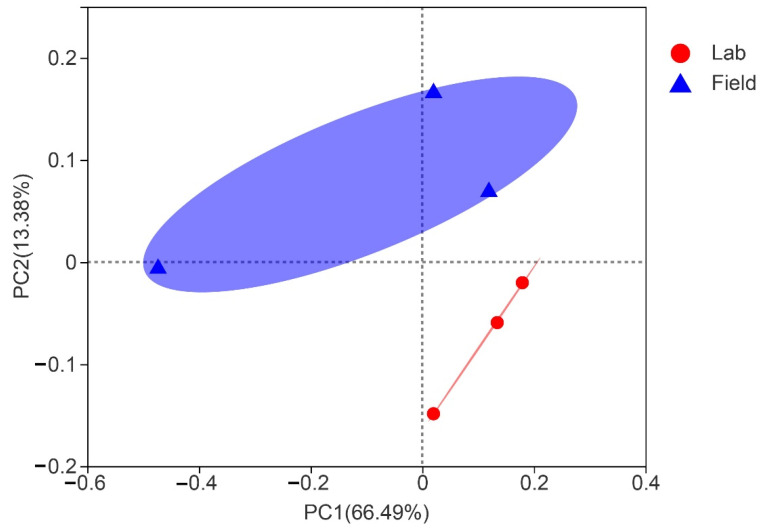
Principal coordinate analysis (PCoA) of community structure from lab and field groups of *S. frugiperda*.

**Figure 4:**
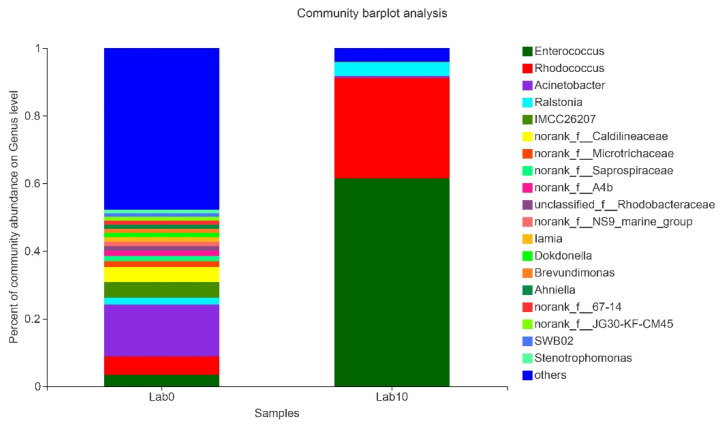
Relative abundance of bacterial composition of *S. frugiperda* after one year of laboratory rearing at the genus level.

The more abundant common OTUs associated with laboratory and field populations of *S. frugiperda* were Moraxellaceae (23.57%), Microtrichaceae (5.25%), Nocardiaceae (4.87%), and Enterococcaceae (4.25%) (Figure S5A). Among the OTUs unique to the laboratory population, the higher contents were Dermatophilaceae (8.33%), Eggerthellaceae (6.25%), Spirochaetaceae (5%), and GEKB124 (4.17%) (Figure S5B). However, among the OTUs unique to the field population, the higher contents were Thermomicrobiaceae (16.49%), Syntrophomonadaceae (7.45%), Neisseriaceae (6.91%), and Cytophagaceae (4.26%) (Figure S5C). We employed Alpha diversity (Shannon’s diversity, Simpson, Chao1, Ace) to estimate the diversity of the microbial community associated with laboratory and field populations of *S. frugiperda.* Alpha diversity analysis showed that there was no significant difference in microbial abundance and diversity between field and laboratory populations (Figure S6). Since the laboratory rearing conditions were stable without various adverse conditions, we explored the changes of gut microbiota in *S. frugiperda* when it was raised in laboratory conditions for more than 10 generations (about one year). The Alpha diversity index showed that both the gut microbiota diversity and community richness of the Lab0 generation were higher than those of the Lab10 generation (Figure S6). PCoA with similar degrees of bacterial communities showed that samples from laboratory populations clustered relatively tightly, but there were significant differences among field population samples (Figure 3). The gut microbial community of Lab0 generation was diverse, while Lab10 generation showed the higher relative proportion of *Enterococcus* and *Rhodococcus* (Figure 4). The similarity analysis results indicated that there were significant differences in the bacterial community of *S. frugiperda* associated with host environment habitat (ANOSIM: R = 0.449, *p* = 0.044; PERMANOVA: R = 0.566, *p* = 0.007).

### 3.5. Comparison of Gut Microbiota of S. frugiperda Fed Maize and Artificial Diet

**Figure 5:**
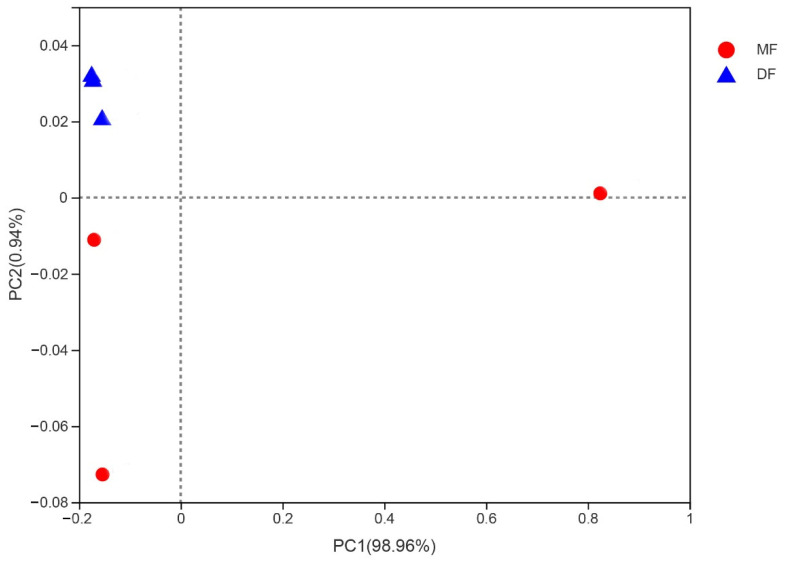
Principal coordinate analysis (PCoA) using the Bray–Curtis dissimilarity measurement comparing the alpha diversity of the bacterial community. MF, *S. frugiperda* fed on maize leaves; DF, *S. frugiperda* fed on artificial diet.

**Figure 6:**
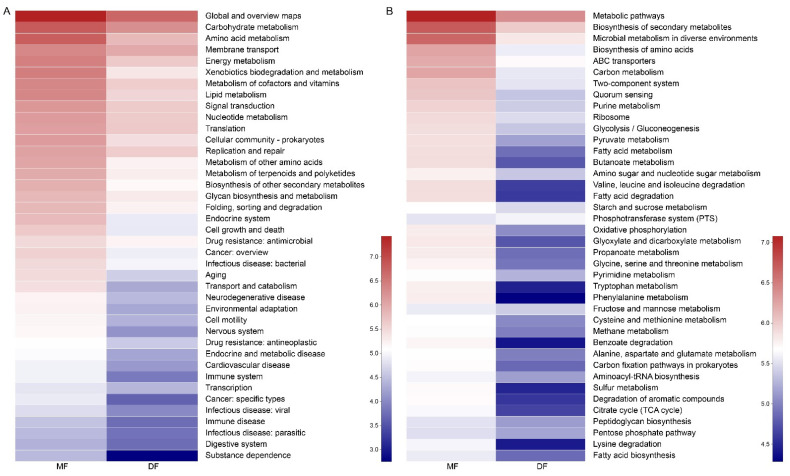
Functional gut microbiota profiles of *S. frugiperda* fed different diets at (**A**) KEGG-level 2 and (**B**) KEGG-level 3.

PCoA analysis using Bray–Curtis indicated that the samples from feeding on artificial diet (DF) were the most uniform, while the samples from feeding on maize (MF) showed higher variation within groups (Figure 5). The Shannon and Simpson indices of the gut microbiota diversity of *S. frugiperda* fed with maize were higher than those fed with artificial diet. The Chao and Ace index suggested a higher community richness of *S. frugiperda* fed on maize compared with that fed on artificial diet (Figure S7). *Enterococcus* and *Rhodococccus* of *S. frugiperda* fed on maize showed the higher relative proportion, while the microbial community of *S. frugiperda* fed on artificial diet was composed mainly of *Enterococcus*, with a total of 98% of the gut microbiota (Figure S8). The similarity analysis results indicated that there were no significant differences in the bacterial community of *S. frugiperda* fed on maize and artificial diet (ANOSIM: R = 0.444, *p* = 0.098; PERMANOVA: R = 0.209, *p* = 0.2). The function of gut microbiota was predicted using the KEGG level 2 and level 3, and the functions such as metabolism, cell growth and death, transport and catabolism, and environmental adaptation were more active in *S. frugiperda* fed on maize (Figure 6).

## 4. Discussion

Systematically analyzing the diversity of microbial communities is challenging due to the high complexity of sampling volume, sampling method, and sampling stage. For example, due to the small size of the eggs and early instar larvae of *S. frugiperda*, a large number of samples is required for sequencing. *S. frugiperda* is a major invasive pest with great reproduction and strong adaptability, which may rely on a variety of microbiota to quickly adapt to different environmental conditions, and such differences may provide a model for investigating and comparing microbial population dynamics. Although microbiomes associated with *S. frugiperda* have been reported in previous studies [23,24], few have investigated the dynamics of microorganisms. In this study, we found support for our hypotheses that *S. frugiperda* utilizes abundant gut microbial community to help it quickly adapt to the environment of the invasion site. Our results indicate that the bacteria in *S. frugiperda* were dominated by Firmicutes and Proteobacteria at the phylum level, which is consistent with previous studies in Lepidopterans [13,35,36,37,38,39]. However, we also found significant differences in the bacterial communities of *S. frugiperda*, which depend on the developmental stages (egg, larvae, and adults), diets, and environmental habitats.

In the present study, we found that *S. frugiperda* differed considerably in the microbial compositions across different life stages. The microbiota diversity was the highest in the egg stage. We speculated that this may be related to the lack of sterilization on the egg surface. When the eggs were sterilized, not enough microorganisms were extracted for sequencing. Therefore, the egg microorganisms might include two parts: most of them were carried by the egg itself, and a few might be the microorganisms in the environment when the egg contacted the environment. The larval gut microbiome was mainly composed of Firmicutes. The results were consistent with the findings of Chen et al. [36] in *Spodoptera littoralis* and Gomes et al. [24] in *S. frugiperda*. Since the food intake of the late larval instars (L4–L6) of *S. frugiperda* was significantly increased compared with that of early larval instars (L1–L3) and the body size grew faster, the changes in the gut microbiota were associated with the growth and development of the host insects, which was consistent with previous reports in *Bombyx mori* [40]. Many studies have shown that early larval stages are more sensitive to environmental changes, which are related to their body sizes and the development of their immune systems [41]. Therefore, the differences in gut microbes between early and late larval stages may also be related to host immunity. *S. frugiperda* is a holometabolous insect, and the gut of adults and larvae have a huge difference. The dynamics of insect gut microbiota can be determined by gut morphology and physicochemical conditions, such as pH and oxygen availability [21,42]. As insects go through their life cycle, gut morphology changes dramatically due to metamorphosis, and gut shape may affect oxygen availability [43,44]. These different gut conditions may lead to changes in the host-specific gut microbiota in insects. Our results showed that gut microbes also were detected in non-feeding adults that had just emerged for one day. Whether these microbes remain before the pupation or exist stably on the gut tissue of *S. frugiperda* requires further research.

Although there were differences in the gut microbiota of *S. frugiperda* during different developmental stages, Firmicutes and Proteobacteria were the dominant bacteria throughout the various developmental stages. The results were consistent with the findings of Broderick et al. [45] in *Lymantria dispar*, Priya et al. [46] in *Helicoverpa armigera*, Xia et al. [35] in *Plutella xylostella* and Chen et al. [40] in *Bombyx mori*. It is known that Proteobacteria and Firmicutes symbionts are involved in the digestion and nutritional utilization of a series of polysaccharides, including cellulose and hemicellulose [47,48,49]. Our results showed that Enterobacteriaceae and Enterococcaceae existed in the whole developmental stage of *S. frugiperda*. Similar to our results, Gomes et al. [24] reported that the dominant bacterium of *S. frugiperda* is Enterococcaceae in five Brazilian states. Enterobacteriaceae contributes to the synthesis of vitamins and pheromones and the degradation of plant compounds, and involves the process of nitrogen fixation and cellulose catabolism [50,51,52]. Enterococcaceae is reported in other Lepidopterous insects such as *Spodoptera litura*, *Manduca sexta*, and *H. armigera* [13,53,54]. *Enterococcus* within Enterococcaceae is able to degrade alkaloids and/or latex, suggesting that *Enterococcus* has a putative role in insect tolerance to their toxic diet [55]. The results obtained above indicate that these conservative bacterial communities could help herbivorous insects adapt to the host and play an important role in physiological metabolism.

Some studies have shown that diet and environment can greatly influence the structure of the host microbiota [56,57]. Our results showed that the diversity of the gut microbes of the laboratory-raised *S. frugiperda* was lower than that directly collected from the field. Correspondingly, the gut microbial diversity of *S. frugiperda* was also reduced after one year of continuous laboratory rearing. The environment of the field is more complex and variable than that of the laboratory, so the *S. frugiperda* may need more symbiotic microorganisms to defend against adverse environments or pathogens. In addition, the leaf microbiome of host plants can be enriched by the environmental microbiome, e.g., by rain splash or wind [58]. Previous studies have shown large differences in microbial titers between field and greenhouse-grown maize leaves [59], which may contribute to differences in gut microbes that were introduced into the gut of *S. frugiperda* through diet consumed.

Previous reports have shown that changing diet can dramatically alter the gut microbiome of the host insect [18,60]. Mason et al. [59] demonstrated that different diets affect the proliferation of gut microbes of *S. frugiperda* by counting colony forming units. Our results by 16S rRNA sequencing suggest that the gut microbiota of *S. frugiperda* fed with maize leaves and artificial diets is differs greatly. On the one hand, since the nutritional components of corn leaves and artificial diets are different, the differences in gut microbial composition of *S. frugiperda* may be related to different nutrient metabolism. A dynamic gut microbiome facilitates adaptation of herbivores to a new diet [61]. On the other hand, maize leaves contain microbes but the artificial diets are sterile, so differences in microbes introduced during feeding may lead to differences in gut microbes. Finally, plant tissues contain large amounts of indigestible and toxic compounds, so herbivorous insects have evolved a range of plant-adaptive strategies, including symbiosis with microbes to adapt to host plants.

In recent years, insect gut microbes have shown great application potential in the development of novel pest biological control strategies, such as *Bacillus thuringiensis* and *Pseudomonas protegens* species [62,63]. Luo et al. [64] reported that *Enterobacter*, *Providencia* and *Serratia* are highly attractive to *Bactrocera tau* adults, which provides a basis for the development of odor attractants made by microorganisms. The invasion of *P. protegens* type strain CHA0 leads to significant changes in gut microbes of *Pieris brassicae*, which eventually results in the death of insect hosts [65]. Therefore, the detailed characterization of the gut microbes of *S. frugiperda* may help to develop novel pest biological control strategies through the elimination of important symbiotic microorganisms or the discovery of entomopathogenic microorganisms.

## 5. Conclusions

The abundant gut microbes of *S. frugiperda* may be beneficial for its abilities of invasion and adaptation. In this study, we collected different *S. frugiperda* gut samples and performed 16S rRNA sequencing. Our results showed that *S. frugiperda* gut microbes vary greatly at different developmental stages and suggest vertical transmission of bacteria in *S. frugiperda*. Furthermore, we found that different environmental conditions and diets can also alter gut microbes. The detailed investigation of the gut microbiota of *S. frugiperda* provides a basis for future research. Since the plasticity of insect gut microbes helps insects utilize different foods and enhances adaptation of insects, a comprehensive understanding of *S. frugiperda*’s gut microbiome will help the development of novel pest control strategies for preventing this invasive pest.

## Data Availability

Raw sequencing data were deposited in the NCBI Short Read Archive (SRA) BioProject PRJNA790707.

## Supplementary Materials

The following supporting information can be downloaded at: https://www.mdpi.com/article/10.3390/insects13090762/s1, Figure S1. Rarefaction curves of each sample based on Miseq sequencing. Figure S2. Comparison of Alpha diversity of gut microbiota across different life stages of *S. frugiperda*. Different letters indicate statistical significance (one-way ANOVA, LSD post hoc test, *p* < 0.05). (**A**) Shannon index; (**B**) Simpson index; (**C**) Chao index; (**D**) Ace index. The larger the Shannon value, the higher the community diversity. The larger the Simpson index value, the lower the community diversity. The larger the Chao and Ace index values, the higher the community richness. Figure S3. Bacterial communities of *S. frugiperda* among different developmental stages. (**A**) Shared bacteria communities between developmental stages; unique bacteria communities to egg (**B**), L1 (**C**), L2 (**D**), L3 (**E**), L4 (**F**), L5 (**G**), L6 (**H**). Figure S4. Gut microbiota of functional profiles of *S. frugiperda* across different life stages at KEGG-level 3. Figure S5. Shared and unique bacterial communities of *S. frugiperda* associated with laboratory and field populations. (**A**) shared bacterial communities of laboratory and field populations; (**B**) unique bacterial communities to laboratory populations and (**C**) field populations. Figure S6. Alpha diversity index of lab and field population of *S. frugiperda*. Different letters indicate statistical significance (one-way ANOVA, LSD post hoc test, *p* < 0.05). (**A**) Shannon index; (**B**) Simpson index; (**C**) Chao index; (**D**) Ace index. Figure S7. Alpha diversity index of *S. frugiperda* fed on different diets (Student’s *t*-test, *p* < 0.05). (**A**) Shannon index; (**B**) Simpson index; (**C**) Chao index; (**D**) Ace index. MF: *S. frugiperda* was reared by maize leaves; DF: *S. frugiperda* was reared by artificial diet. Figure S8. Relative abundance of gut bacterial community of *S. frugiperda* related to different diets at the genus level. Table S1. Primers used in this study. Table S2. Sequencing data statistics of all samples. Table S3. Bacteria identified persist throughout different stages of development.
