# Supplementary material for: Fall Armyworm Gut Bacterial Diversity Associated with Different Developmental Stages, Environmental Habitats, and Diets
Source: Insects. 2022 Aug 24;13(9):762. doi: 10.3390/insects13090762 (PMC9503601; doi:10.3390/insects13090762)
Supplement: Supplementary file 1 [file insects-13-00762-s001.zip › Table S1.pdf]

Table S1. Primers used in this study.

Software/database	Version	Application	Link
Flash	1.2.11	pair-end sequence splicing	https://ccb.jhu.edu/software/FLASH/index.shtml
Qiime	1.9.1	Generate abundance table for each taxonomy level, calculate beta diversity distance	http://qiime.org/install/index.html
Uparse	7.0.1090	OTU cluster	http://www.drive5.com/uparse/
RDP Classifier	2.1	Sequence classification annotation	https://sourceforge.net/projects/rdp-classifier/
Usearch	7.0	OTU statistics	http://www.drive5.com/usearch/
Mothur	1.30.2	alpha diversity analysis	https://www.mothur.org/wiki/Download_mothur
PICRUSt	1.1.0	function prediction of KEGG、COG、Pfam	http://picrust.github.io/picrust/
Mega	7.0	phylogenetic analysis	https://www.megasoftware.net/
SILVA	132	rRNA database	https://www.arb-silva.de/
RDP	11.5	rRNA database	http://rdp.cme.msu.edu/
GreenGenes	135	rRNA database	http://greengenes.secondgenome.com/
FunGene	9.6	Functional gene database	http://www.fungene-db.fr/
Tax4fun	0.3.1	Tax4Fun function prediction	http://tax4fun.gobics.de/
MAFFT	7.2	Multiple sequence alignment	https://mafft.cbrc.jp/alignment/software/
IQ-TREE	1.6.8	Constructing evolutionary trees	http://www.iqtree.org/
Fastp	0.19.6	Quality control	https://github.com/OpenGene/fastp
