# Supplementary material for: Fall Armyworm Gut Bacterial Diversity Associated with Different Developmental Stages, Environmental Habitats, and Diets
Source: Insects. 2022 Aug 24;13(9):762. doi: 10.3390/insects13090762 (PMC9503601; doi:10.3390/insects13090762)
Supplement: Supplementary file 1 [file insects-13-00762-s001.zip › Table S2.pdf]

Table S2 Sequencing data statistics of all samples

Sample\Info	Seq_num	Base_num	Mean_length	Min_length	Max_length
Egg_1	33972	14452598	425.4268	264	437
Egg_2	33861	14469972	427.3345	209	431
Egg_3	50598	21495716	424.8333	239	437
L1_1	44903	19262729	428.9853	403	430
L1_2	44870	19246791	428.9456	248	430
L1_3	35970	15423748	428.7948	209	518
L2_1	52622	22574259	428.989	404	430
L2_2	43056	18470703	428.9925	404	489
L2_3	42328	18128173	428.2785	249	444
L3_1	37810	16219358	428.9701	404	430
L3_2	42468	18217473	428.9694	347	432
L3_3	36896	15826941	428.9609	403	527
L4_1	36624	14745866	402.6285	237	498
L4_2	31451	13458842	427.9305	403	431
L4_3	48883	20958596	428.7502	403	430
L5_1	33262	14255175	428.5724	388	430
L5_2	41419	16985799	410.0968	259	498
L5_3	52434	22485103	428.8268	403	432
L6_1	41407	17514672	422.9882	200	521
L6_2	46815	19822978	423.4322	202	495
L6_3	88537	37911890	428.2039	400	431
Female_1	38448	16493708	428.9874	277	430
Female_2	41404	17761878	428.9894	404	430
Female_3	36934	15843982	428.9809	403	430
Male_1	41786	17924601	428.9619	248	431
Male_2	36348	15592312	428.973	404	430
Male_3	39632	17001280	428.9786	200	430
Lab_1	57566	23942432	415.9127	262	528
Lab_2	48283	20118635	416.6815	264	479
Lab_3	41594	17419696	418.8031	204	478
Field_1	53373	22305692	417.9209	389	479
Field_2	47805	19929785	416.8975	224	470
Field_3	52542	22375862	425.8662	201	505
DF_1	30861	13228497	428.6477	262	431
DF_2	39444	16918849	428.9334	262	430
DF_3	39552	16967418	428.9901	406	431
MF_1	44881	18251547	406.6653	233	499
MF_2	45712	18591287	406.7047	227	446
MF_3	40683	16523741	406.1584	212	439
