# Supplementary material for: Fall Armyworm Gut Bacterial Diversity Associated with Different Developmental Stages, Environmental Habitats, and Diets
Source: Insects. 2022 Aug 24;13(9):762. doi: 10.3390/insects13090762 (PMC9503601; doi:10.3390/insects13090762)
Supplement: Supplementary file 1 [file insects-13-00762-s001.zip › Table S3.pdf]

Table S3 Bacterial identified persist throughout different stages of development

OUT ID	Phylum	Genus
OTU478	Proteobacteria	Enterobacter
OTU956	Actinobacteriota	Corynebacterium
OTU877	Proteobacteria	-
OTU346	Firmicutes	Enterococcus
OTU784	Firmicutes	Enterococcus
OTU884	Proteobacteria	Acinetobacter
