# Supplementary figures and images for: Fall Armyworm Gut Bacterial Diversity Associated with Different Developmental Stages, Environmental Habitats, and Diets
Source: Insects. 2022 Aug 24;13(9):762. doi: 10.3390/insects13090762 (PMC9503601; doi:10.3390/insects13090762)

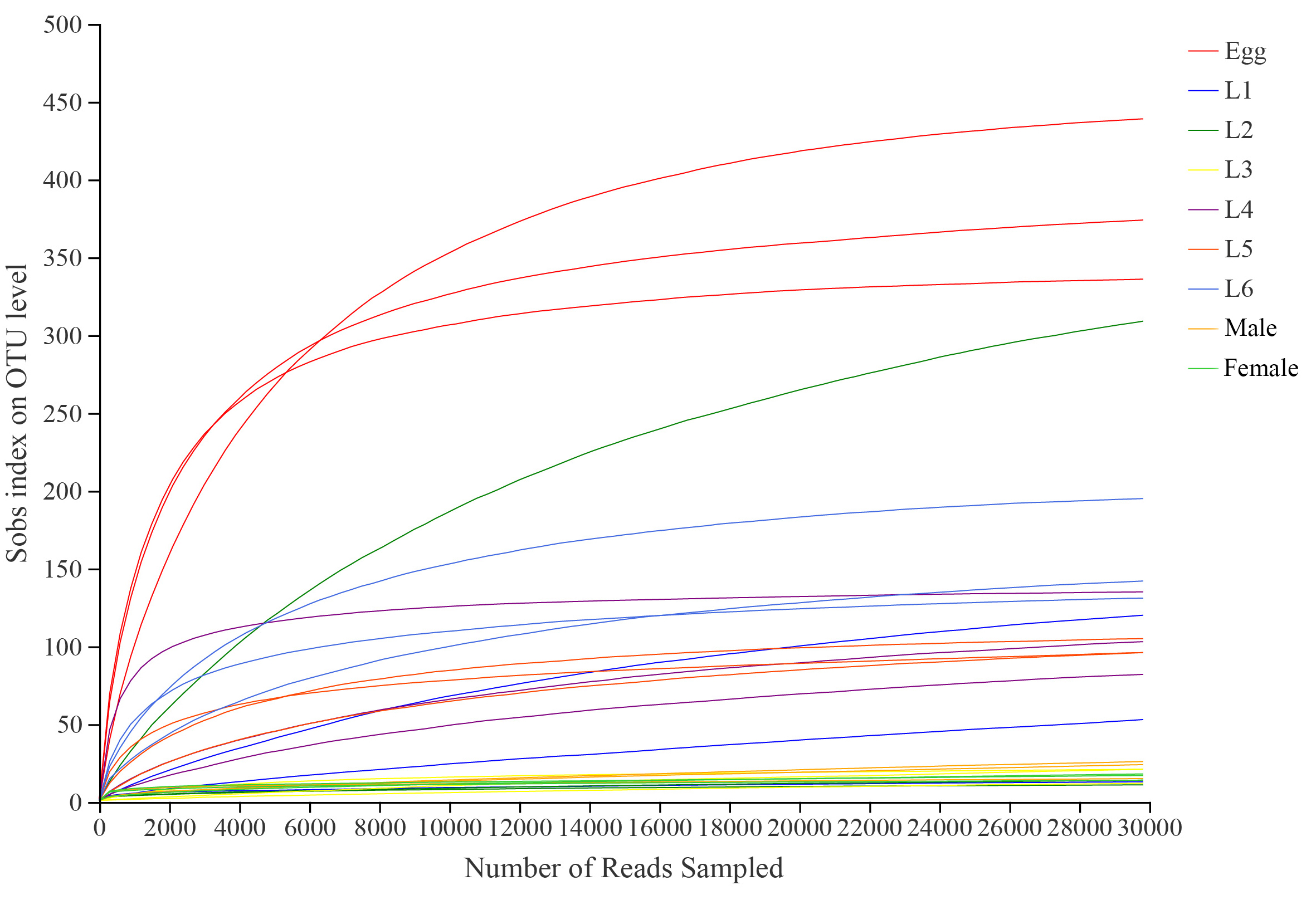

Supplement: Supplementary file 1 [file insects-13-00762-s001.zip › Figure S1.jpg]

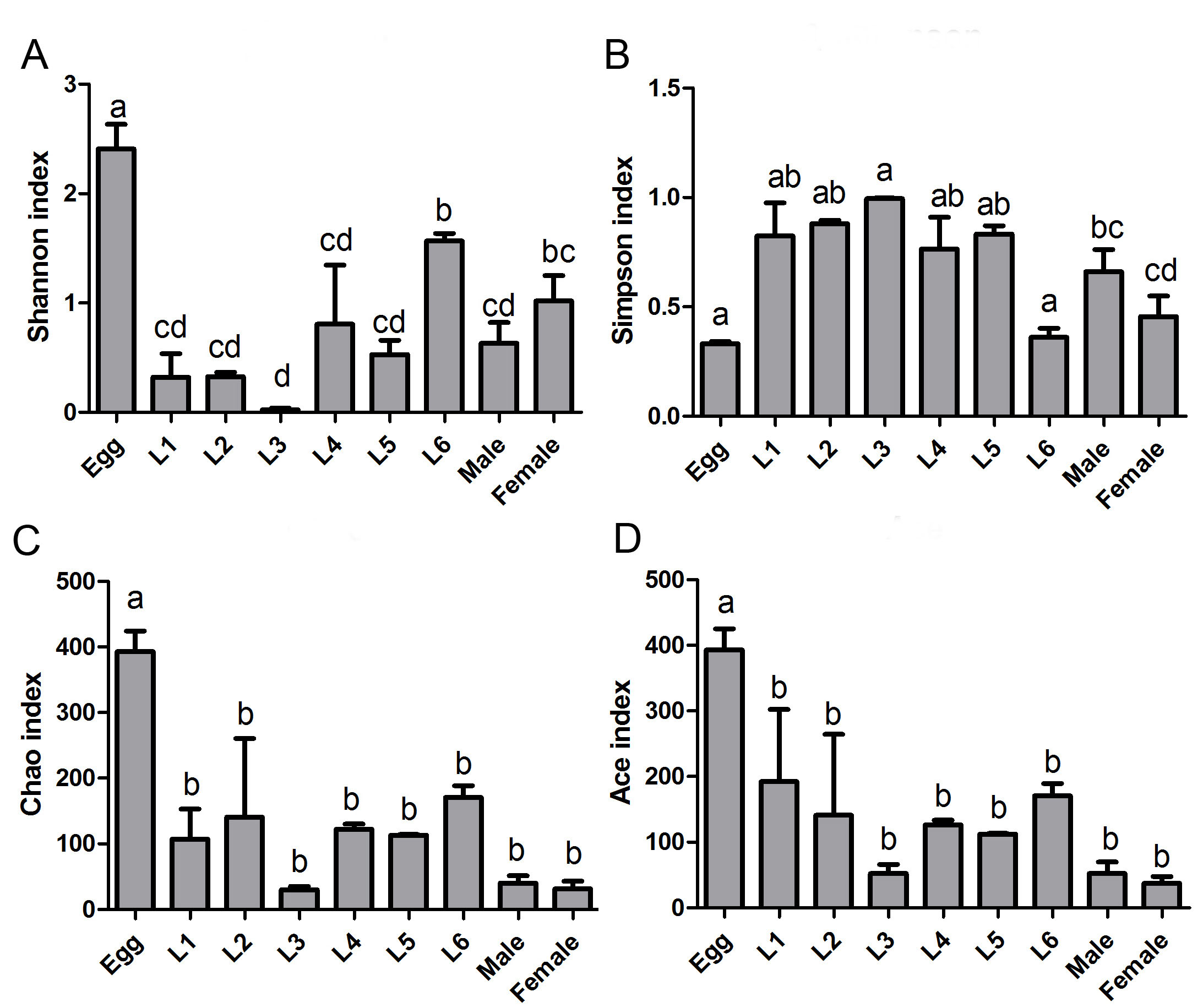

Supplement: Supplementary file 1 [file insects-13-00762-s001.zip › Figure S2.jpg]

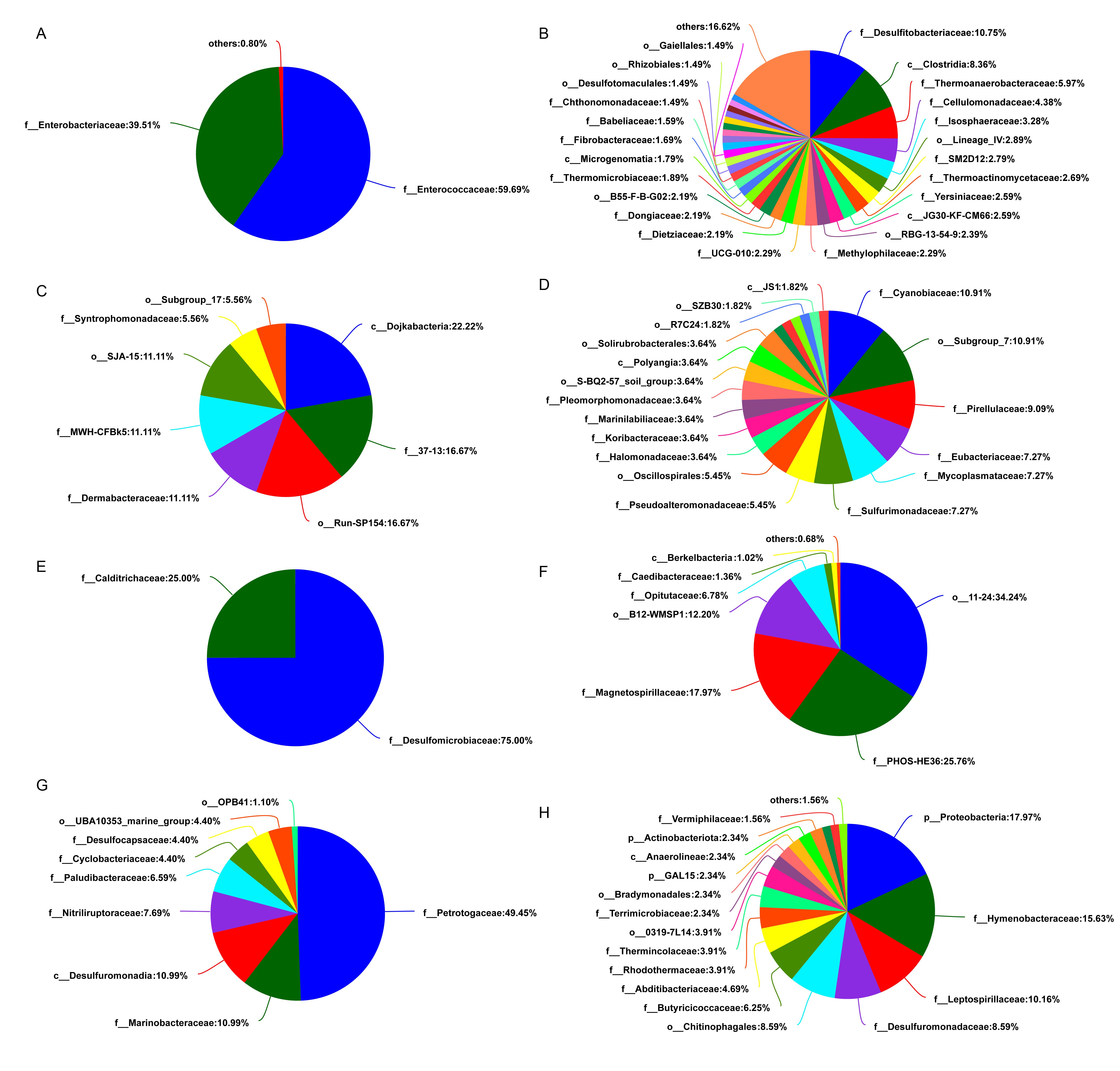

Supplement: Supplementary file 1 [file insects-13-00762-s001.zip › Figure S3.jpg]

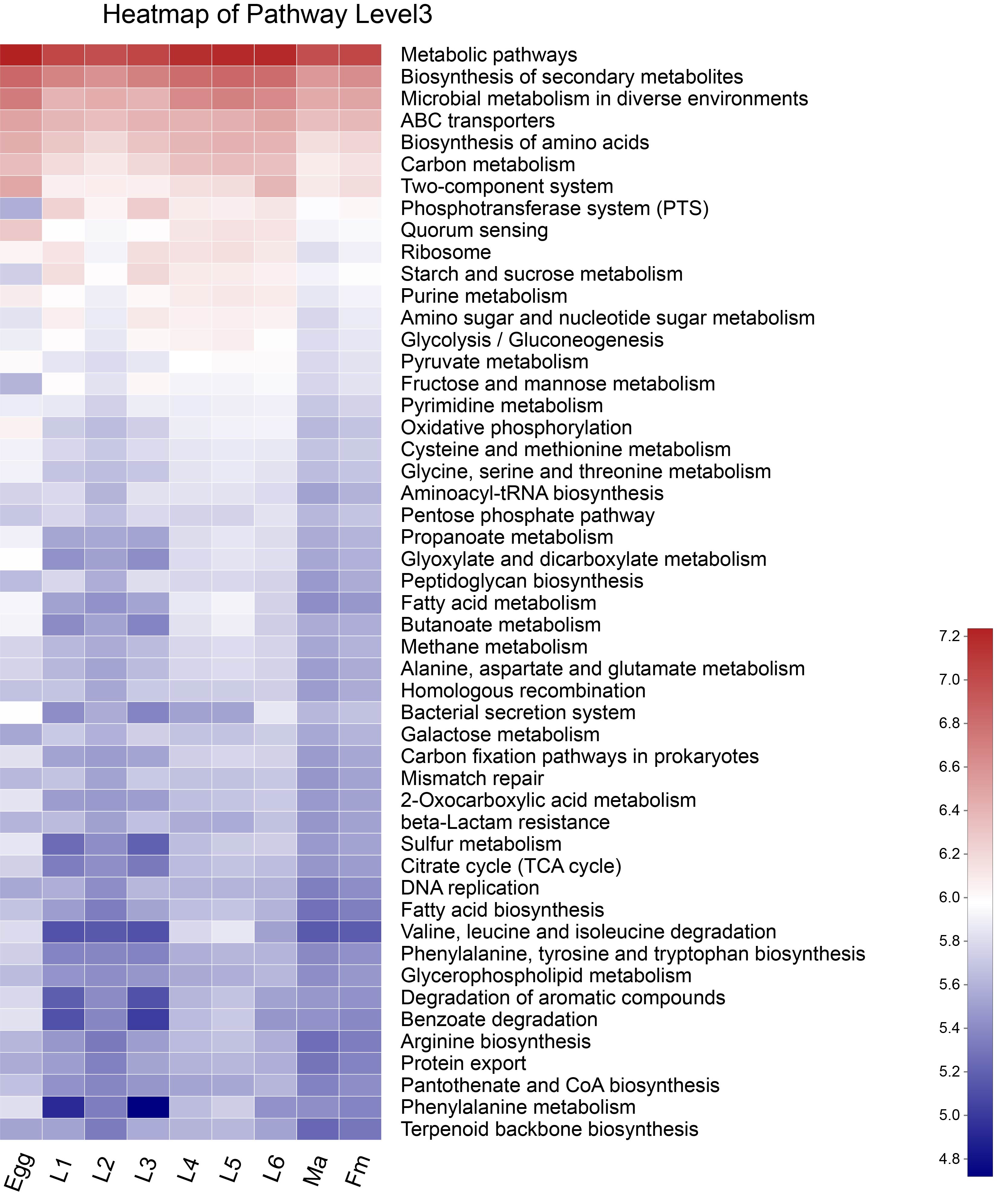

Supplement: Supplementary file 1 [file insects-13-00762-s001.zip › Figure S4.jpg]

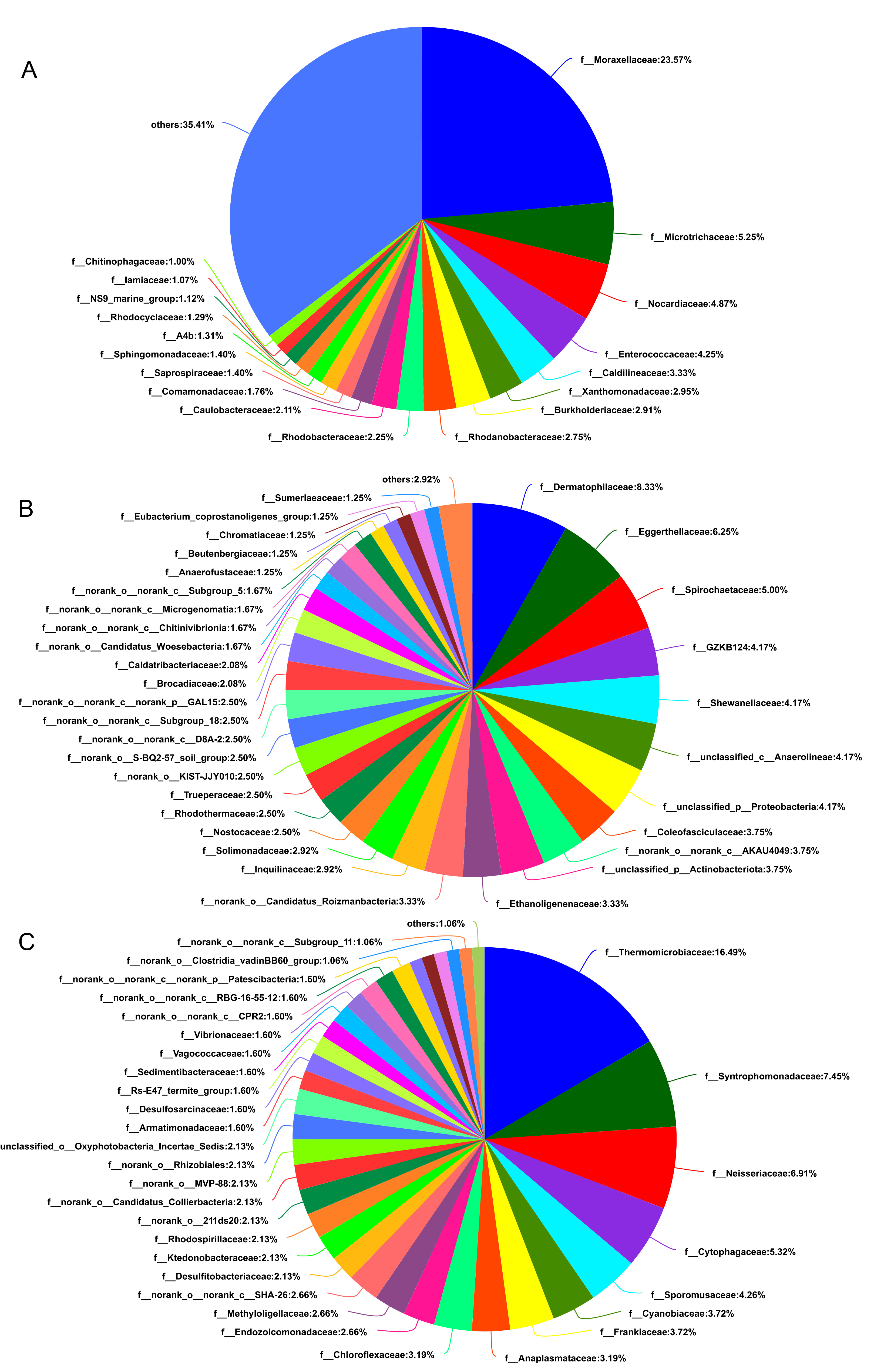

Supplement: Supplementary file 1 [file insects-13-00762-s001.zip › Figure S5.jpg]

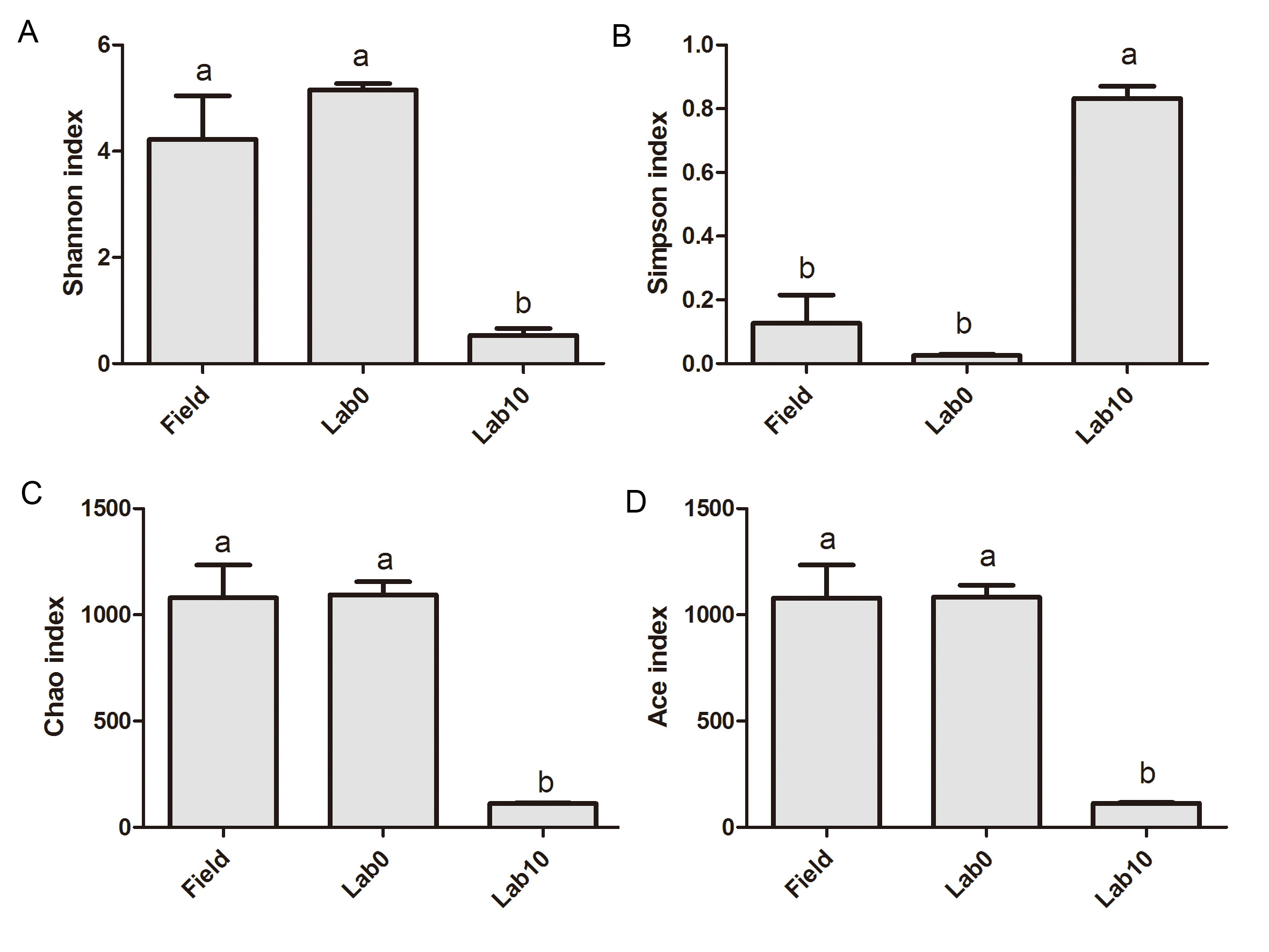

Supplement: Supplementary file 1 [file insects-13-00762-s001.zip › Figure S6.jpg]

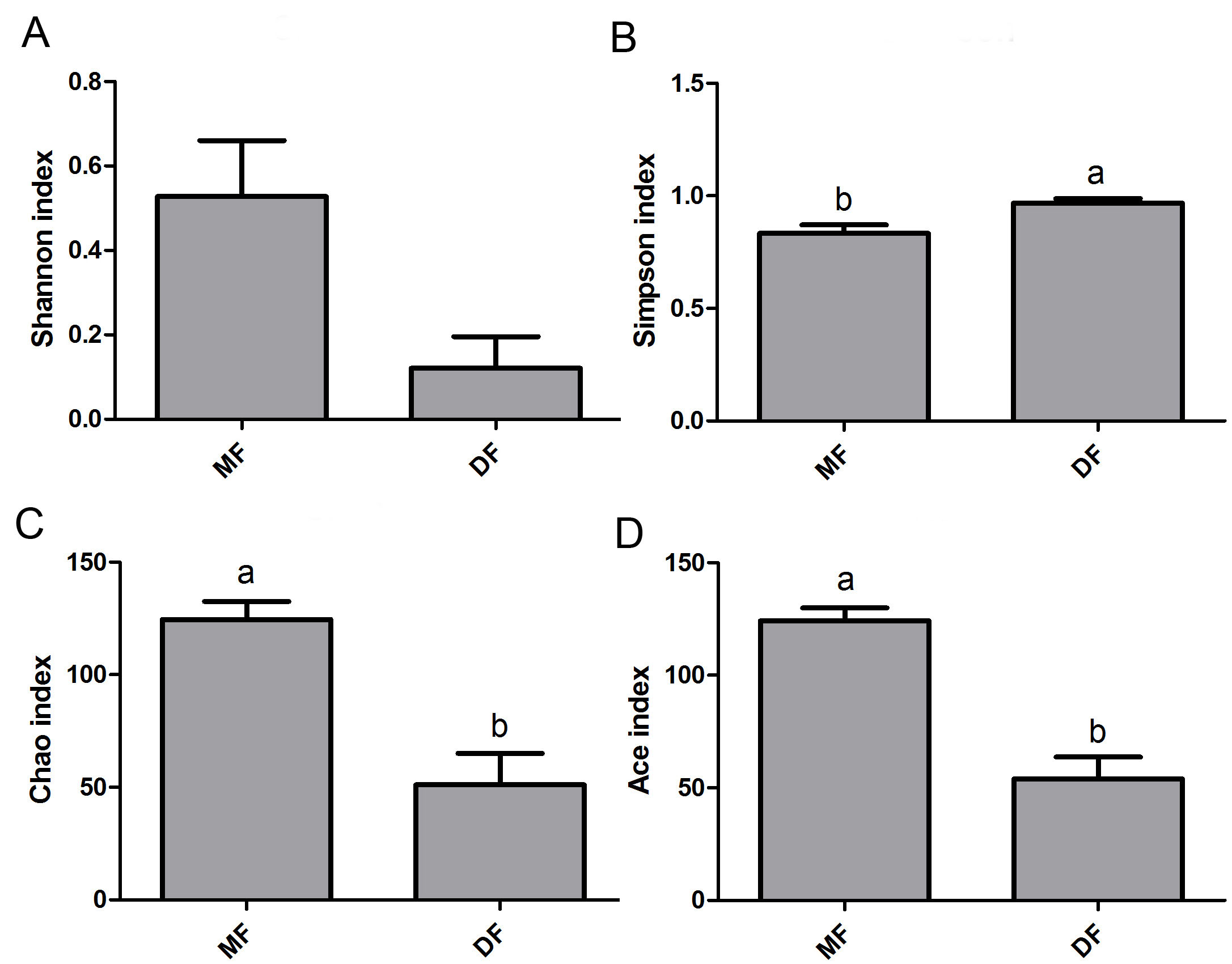

Supplement: Supplementary file 1 [file insects-13-00762-s001.zip › Figure S7.jpg]

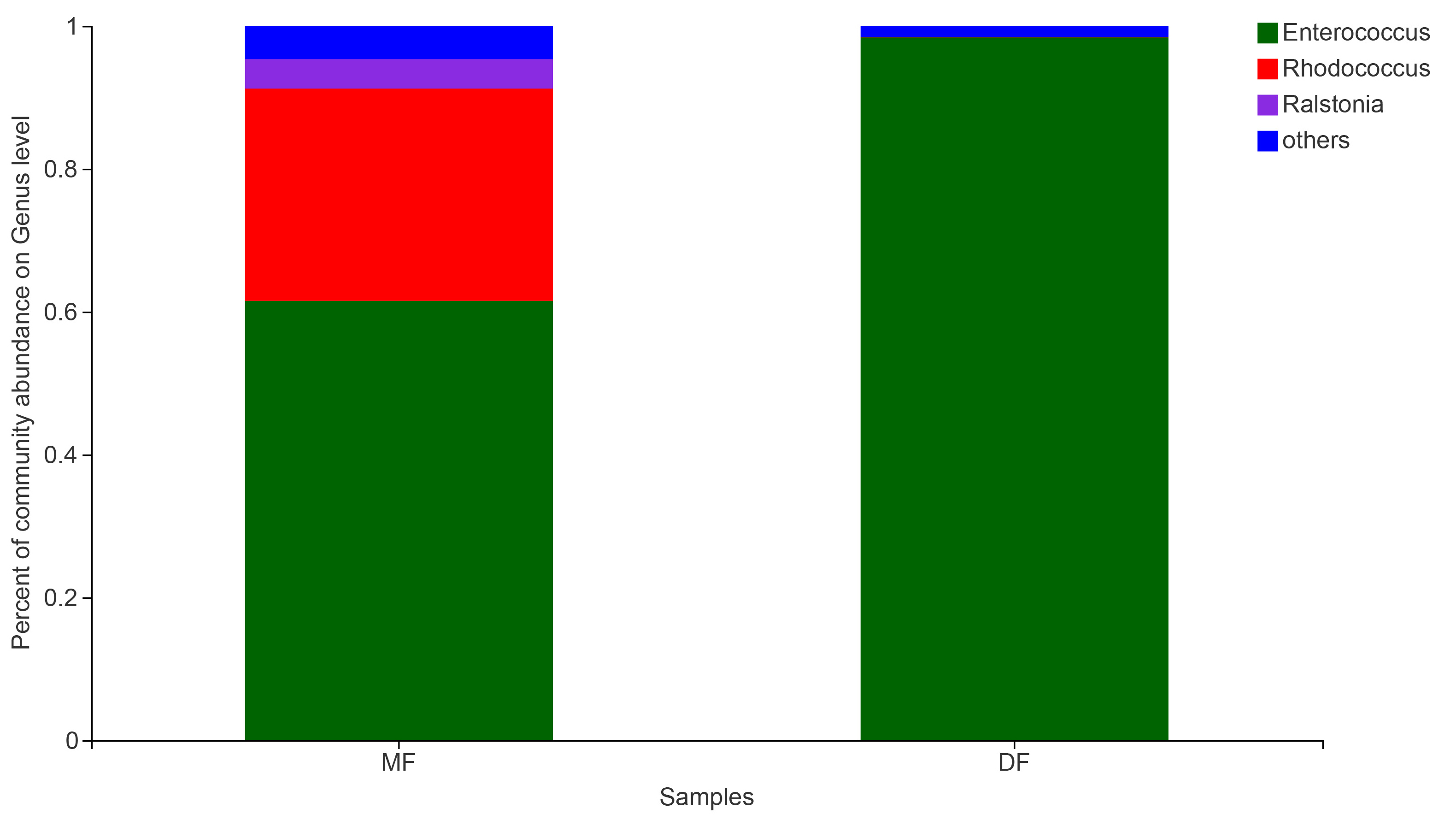

Supplement: Supplementary file 1 [file insects-13-00762-s001.zip › Figure S8.jpg]
